# Antimicrobial and Antibiofilm Activity of the Probiotic Strain *Streptococcus salivarius* K12 against Oral Potential Pathogens

**DOI:** 10.3390/antibiotics10070793

**Published:** 2021-06-29

**Authors:** Andrea Stašková, Miriam Sondorová, Radomíra Nemcová, Jana Kačírová, Marián Maďar

**Affiliations:** 1 Clinic of Stomatology and Maxillofacial Surgery, Faculty of Medicine, University of Pavol Jozef Šafárik in Košice, 040 01 Košice, Slovakia; andrea.staskova@icloud.com; 2 Department of Microbiology and Immunology, University of Veterinary Medicine and Pharmacy in Košice, 041 81 Košice, Slovakia; radomira.nemcova@uvlf.sk (R.N.); kacirova.jana@gmail.com (J.K.); madarmarian@gmail.com (M.M.)

**Keywords:** oral probiotic, *Streptococcus salivarius* K12, cell-free supernatant, antimicrobial activity, antibiofilm activity, oral pathogens

## Abstract

Oral probiotics are increasingly used in the harmonization of the oral microbiota in the prevention or therapy of various oral diseases. Investigation of the antimicrobial activity of the bacteriocinogenic strain *Streptococcus salivarius* K12 against oral pathogens shows promising results, not only in suppressing growth, but also in eliminating biofilm formation. Based on these findings, we decided to investigate the antimicrobial and antibiofilm activity of the neutralized cell-free supernatant (nCFS) of *S. salivarius* K12 at various concentrations against selected potential oral pathogens under in vitro conditions on polystyrene microtiter plates. The nCFS of *S. salivarius* K12 significantly reduced growth (*p* < 0.01) in *Streptococcus mutans* Clarke with increasing concentration from 15 to 60 mg/mL and also in *Staphylococcus hominis* 41/6 at a concentration of 60 mg/mL (*p* < 0.001). Biofilm formation significantly decreased (*p* < 0.001) in *Schaalia odontolytica* P10 at nCFS concentrations of 60 and 30 mg/mL. Biofilm inhibition (*p* < 0.001) was also observed in *Enterobacter cloacae* 4/2 at a concentration of 60 mg/mL. In *Schaalia odontolytica* P10 and *Enterobacter cloacae* 4/2, the nCFS had no effect on their growth.

## 1. Introduction

Bacteria are the predominant microorganisms in the resident oral microbiota. The diversity of bacterial species in the oral cavity is influenced not only by the availability of nutrients but also by the ability to survive in the form of a biofilm, depending on its location (surface of teeth, tongue, and mucous membranes). The resident oral microbiota competes and excludes exogenous pathogens and also contributes to the normal development of tissues and the immune system [1,2]. However, the homeostatic balance between host and microbial communities can be disturbed by many influences, which can lead to the development of oral diseases [3], such as dental caries, gingivitis, periodontitis [4], pharyngotonsillitis, and others [5]. The cause of these diseases is mostly pathogenic bacteria found in the oral cavity. Potential oral pathogenic bacteria are able to form a biofilm, and such bacteria include *Streptococcus mutans* [6], *Schaalia odontolytica* [7], *Staphylococcus hominis* [8,9], and *Enterobacter cloacae* [10].

Interest in applying new strategies for the treatment of diseases, such as probiotics, is rising significantly due to increasing antibiotic resistance [11]. The living beneficial microorganisms found in probiotic preparations provide a health benefit to the host, either in maintaining or in improving the microbiota [12]. Potentially pathogenic microorganisms enter the body through the mouth or nose, which is why oral probiotics are an excellent first line of defense of the mouth and throat. Oral probiotics have the potential to modify the oral microbiota, thereby helping to prevent or treat oral diseases. They prevent the formation of dental plaque, promote the health of gums and teeth, and prevent halitosis [13,14]. Commonly used oral probiotic bacteria include strains of *Lactobacillus reuteri* ATCC 55730/ATCC PTA 5289, *Lactobacillus rhamnosus* GG, and *Streptococcus salivarius* K12 [15].

The oral cavity of newborns is initially highly selective for bacteria and therefore allows colonization by only a few bacterial species. *S. salivarius* is one of the first and most important pioneer colonizers of the epithelial surfaces of the oral cavity and upper respiratory tract of humans. It remains there as the predominant member of the oral microbiota throughout life [16,17,18,19]. *S. salivarius* strains inhibit the formation of a biofilm of *S. mutans*, which is involved in dental caries, and suppress potentially pathogenic bacteria in the upper respiratory tract, e.g., *Streptococcus pneumoniae* and *Streptococcus pyogenes* in vitro [20]. The *S. salivarius* K12 strain, which has been isolated from the saliva of a healthy child, produces a variety of antimicrobial peptides and is one of the first commercially available oral probiotics [21,22]. The oral probiotic strain *S. salivarius* K12 has therapeutic potential in pharyngo-tonsillar infections caused by *S. pyogenes* [23] and in the treatment of halitosis. It has been shown to inhibit *Solobacterium moorei*, *Eubacterium saburreum*, *Streptococcus anginosus*, and *Parvimonas micra* (formerly *Micromonas micros*), which are involved in oral malodor [24,25]. It also has a beneficial effect on reducing otitis media [26]. *S. salivarius* K12 may play a protective role against oral candidiasis. It has not been shown to be directly fungicidal but appears to inhibit *Candida* adhesion by preferential binding to hyphae [27].

The aim of the present study was to investigate the in vitro antimicrobial and antibiofilm activity of various concentrations of a neutralized cell-free supernatant (nCFS) from the probiotic strain *S. salivarius* K12 against potential oral pathogens.

## 2. Results

### 2.1. Identification of S. salivarius K12

BLASTn analysis within the 16S rRNA variable region of the *S. salivarius* K12 isolate obtained from Bactoral confirmed its identity with *S. salivarius* at 99.72%. Genotyping based on specific primers for the *gtf* gene also confirmed a consensus sequence (543 bp) identical to *S. salivarius*. The genes encoding salivaricin A (99.67% identity) and B (100% identity) were detected in this isolate. The results show that the isolated strain from the commercially available Bactoral preparation was successfully confirmed as *S. salivarius* K12.

### 2.2. Antimicrobial and Antibiofilm Activity of the Neutralized Cell-Free Supernatant of S. salivarius K12 against Potential Oral Pathogens

Potentially oral pathogenic strains, namely *S. mutans* Clarke, *Staphylococcus hominis* 41/6, *Enterobacter cloacae* 4/2, and *Schaalia odontolytica* P10, were used to test the antimicrobial and antibiofilm activity of various concentrations of the nCFS of *S. salivarius* K12. Biofilm formation was previously confirmed in the tested strains.

**Figure 1:**
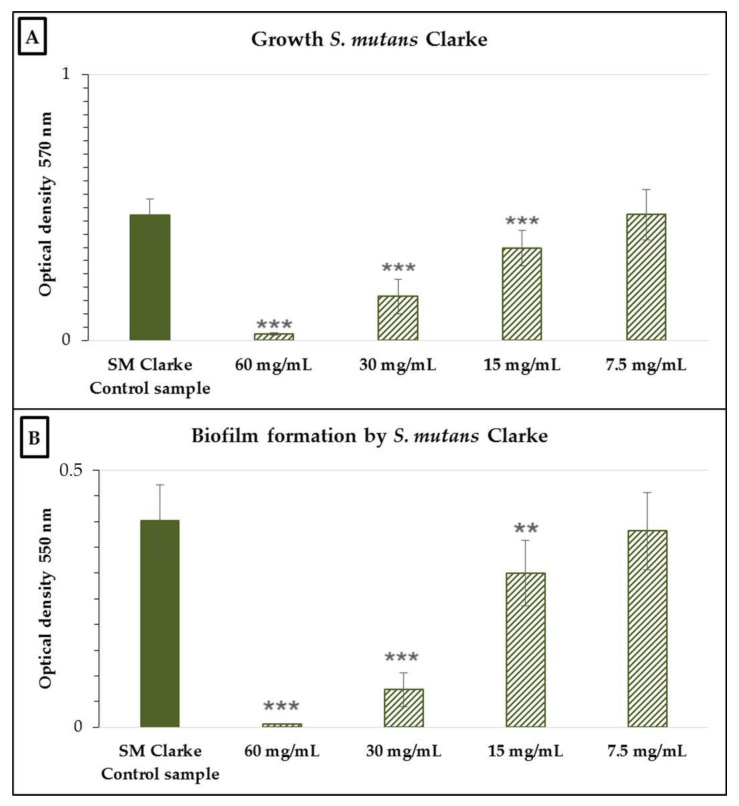
Growth (**A**) and biofilm formation (**B**) of *Streptococcus mutans* Clarke in the presence of various concentrations of the neutralized cell-free supernatant of *Streptococcus salivarius* K12. SM Clarke control sample: *S. mutans* Clarke in BHI without the nCFS of *S. salivarius* K12. Data are expressed as the arithmetic mean ± standard deviation; *** *p* < 0.001 and ** *p* < 0.01 compared to the control.

The nCFS of *S. salivarius* K12 had an inhibitory effect on growth, which subsequently affected the biofilm formation of *S. mutans* Clarke (Figure 1). Its growth decreased significantly (*p* < 0.001) compared to the control with increasing concentration from 15 to 60 mg/mL. At a concentration of 7.5 mg/mL, there was no significant inhibitory effect on either growth or biofilm formation. The percentage inhibition of growth was 87.48 ± 1.01, 64.32 ± 3.67, and 27.31 ± 5.23% at a concentration of 60, 30, and 15 mg/mL, respectively.

**Figure 2:**
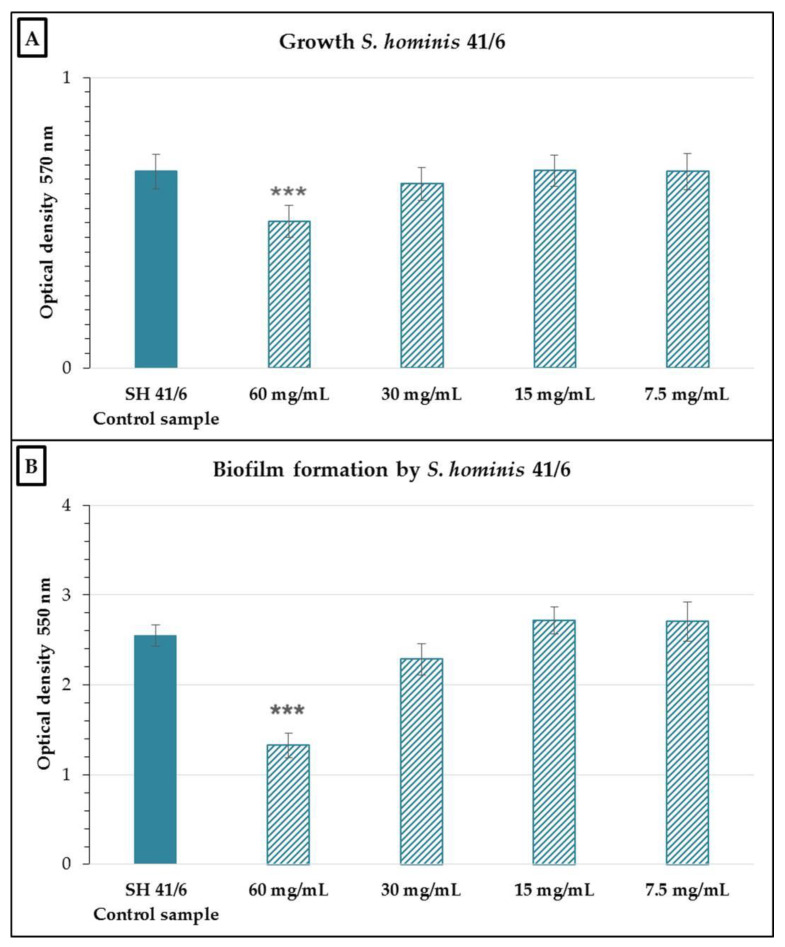
Growth (**A**) and biofilm formation (**B**) of *Staphylococcus hominis* 41/6 in the presence of various concentrations of the nCFS of *S. salivarius* K12. SH 41/6 control sample: *S. hominis* 41/6 in BHI without the nCFS of *S. salivarius* K12. Data are expressed as the arithmetic mean ± standard deviation; *** *p* < 0.001 compared to the control.

Analysis of growth and biofilm formation in *S. hominis* 41/6 in the presence of various concentrations of the nCFS showed a significant effect compared to the control (*p* < 0.001) only at a concentration of 60 mg/mL (Figure 2). In terms of percentage, this represented a 23.16% ± 4.37% growth inhibition and a 42.59% ± 5.07% inhibition of biofilm formation at this concentration.

**Figure 3:**
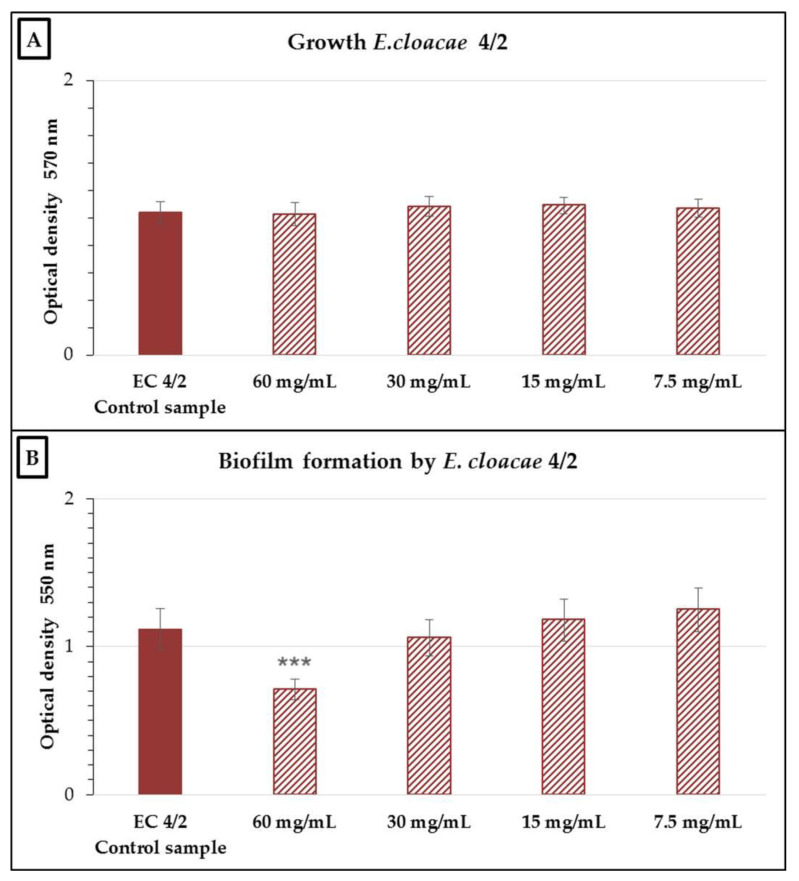
Growth (**A**) and biofilm formation (**B**) of *Enterobacter cloacae* 4/2 in the presence of various concentrations of the nCFS of *S. salivarius* K12. EC 4/2 control sample: *E. cloacae* 4/2 in BHI without the nCFS of *S. salivarius* K12. Data are expressed as the arithmetic mean ± standard deviation; *** *p* < 0.001 compared to the control.

None of the nCFS concentrations tested significantly inhibited the growth of the *E. cloacae* 4/2 strain (Figure 3). In the evaluation of antibiofilm activity, there was a significant reduction (*p* < 0.001) in biofilm formation compared to the control at a concentration of 60 mg/mL, which represented a 32.37% ± 3.7% inhibition of biofilm formation.

**Figure 4:**
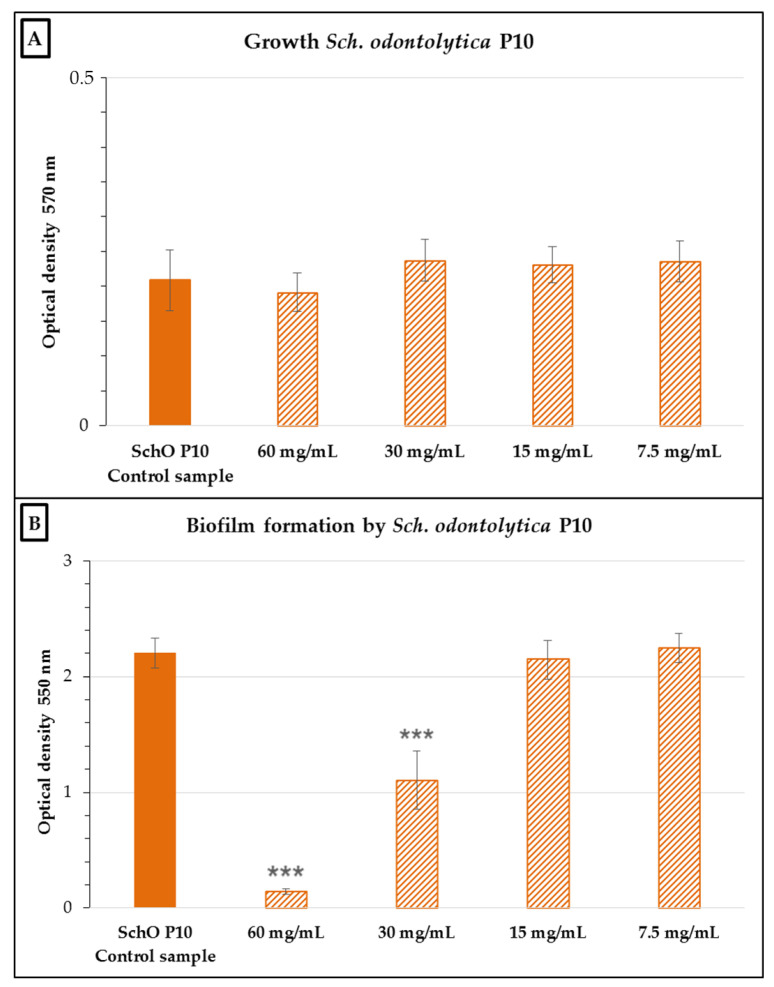
Growth (**A**) and biofilm formation (**B**) of *Schaalia odontolytica* P10 in the presence of various concentrations of the nCFS of *S. salivarius* K12. SchO P10 control sample: *Sch. odontolytica* P10 in BHI without the nCFS of *S. salivarius* K12. Data are expressed as the arithmetic mean ± standard deviation; *** *p* < 0.001 compared to the control.

Similar to *E. cloacae* 4/2, the nCFS had no significant inhibitory effect on the growth of the *Sch. odontolytica* P10 strain (Figure 4). Testing of the inhibitory activity of the nCFS against *Sch. odontolytica* P10 showed a significant effect (*p* < 0.001) on the reduction in biofilm formation at concentrations of 60 and 30 mg/mL compared to the control. The biofilm inhibition was 91.03% ± 1.09% and 48.56% ± 4.48% at concentrations of 60 and 30 mg/mL, respectively.

## 3. Discussion

In the present study, the CFS of *S. salivarius* K12 was investigated against selected oral potential pathogens. Neutralization of the supernatant eliminated the possible effect of acidic products on the inhibition of the tested strains, and its antimicrobial and antibiofilm activity was also demonstrated. The most commonly used method for testing antimicrobial and antibiofilm activity is based on the use of 96-well microtiter plates [28]. Yoo et al. [29] evaluated the antimicrobial activity of the supernatant of oral probiotic strains *S. salivarius* K12 and M18 in wells of a 96-well polystyrene culture plate against *Porphyromonas gingivalis* and *Treponema denticola*. Antimicrobial activity above a certain concentration level has been reported. In this study, a standard assay in microtiter plates was used to examine in vitro antimicrobial and antibiofilm activity, similar to previous studies [29,30]. In addition to the standard methodology, new innovative approaches to in vitro testing of antimicrobial activity with inhibition of biofilm formation are also available, such as the dental implant in vitro model stimulating biofilm formation [31], the in vitro lab catheterization model [32], and biofilm formation in real time using automated microfluidics [28].

*S. salivarius* strains inhibit biofilm formation by *S. mutans* and *S. pyogenes* [33]. In the present study, inhibition of both growth and biofilm formation of strains of *S. mutans* Clarke and *S. hominis* 41/6 was noted. *S. mutans* was more sensitive even at lower concentrations of the nCFS compared with *S. hominis*. *S. mutans* is considered the major etiological agent of dental caries and, due to virulence factors, such as glucan production, acid resistance, natural competence, and compact biofilm formation, has a certain advantage over other oral bacteria [34]. Ogawa et al. [30] identified inhibitors produced by *S. salivarius* HT9R, JCM5707, and ATCC 9759 strains, which prevented biofilm formation in *S. mutans* GS-5 on the surface of saliva-coated 96-well polystyrene plates and hydroxyapatite supplemented with sucrose. Based on protein analysis, the biofilm-inhibiting agent was identified as the enzyme exo-beta-D-fructosidase (FruA). The results suggest that *S. salivarius* FruA may modulate sucrose-dependent colonization of *S. mutans* on oral surfaces. FruA is produced not only by *S. salivarius* strains but also by other oral streptococci. FruA may play a role in the formation of oral biofilms by bacteria and may regulate microbial pathogenicity in the oral cavity. Hyink et al. [35] identified in *S. salivarius* K12 a heat-stable inhibitor active against *Enterococcus faecalis* ATCC 19433 and *Actinomyces naeslundii* NCTC 10301 and a heat-labile inhibitor active against *S. hominis* 2203. Frickmann et al. [36] found that the CFS of *S. salivarius* K12, in addition to reducing and preventing biofilm formation in *Staphylococcus epidermidis* (ATCC 35984), also affects the dispersion of the already pre-formed biofilm. The CFS of *S. salivarius* 24SMB and *Streptococcus oralis* 89a strains isolated from the commercial preparation reduced biofilm formation in vitro and was able to eradicate the pre-formed biofilm of typical pathogenic respiratory bacteria, such as *S. pyogenes*, *S. pneumoniae*, *Moraxella catarrhalis*, *Staphylococcus aureus*, *S. epidermidis*, and *Propionibacterium acnes* [37].

*S. salivarius* K12 produces the bacteriocins salivaricin A2 and B belonging to the group of lantibiotics [38]. Lantibiotics show two mechanisms of action and mainly have a broad spectrum of activity against Gram-positive bacteria. They inhibit the synthesis of peptidoglycan and are involved in the formation of pores in the cytoplasmic membrane [39]. Salivaricin B differs from salivaricin A by its mechanism of action in interfering with cell wall biosynthesis [40]. Megaplasmid-encoding salivaricin A and salivaricin B carried by the probiotic strain *S. salivarius* K12 may be transmissible between *S. salivarius* strains in vitro and in vivo [41]. We hypothesize that salivaricins and their antagonistic effect on strain growth may be involved in the observed inhibitory effect on biofilm formation in *S. mutans* and *S. hominis*.

Vacca et al. [31] modeled the interaction between a pathogenic biofilm-forming strain *Streptococcus intermedius* DSMZ 20573 and a probiotic bacteriocin-producing strain *S. salivarius* K12. *S. intermedius* was co-cultured with *S. salivarius* K12 in vitro in a model simulating biofilm formation in a dental implant composed of a titanium cylindrical surface. Biofilm formation was assessed by quantifying the number of bacteria and the expression level of the luxS gene. The authors recorded an 87% reduction in *S. intermedius* numbers in the biofilm. In addition, the reduction was accompanied by reduced expression of the luxS gene involved in biofilm formation in *S. intermedius*.

*Sch. odontolytica*, also known as *Actinomyces odontolyticus*, is considered an early colonizer, adhering directly to the salivary pellicle coating the tooth surface [42]. *Sch. odontolytica* was not generally detected in healthy patients, but it was isolated from persons with advanced dental caries and root canal infections [43]. *E. cloacae* is not usually found in a healthy oral microbiota but has been most often isolated in adults with advanced periodontitis [44]. In addition, *E. cloacae* is reported as an important opportunistic and multiresistant bacterial pathogen for humans in hospital wards [45]. In *Sch. odontolytica* P10 and *E. cloacae* 4/2 strains, inhibition of biofilm formation was observed without affecting their growth. This may involve influencing the expression of genes involved in the synthesis of adhesins responsible for adherence, i.e., in the initial phase of biofilm formation or in intercellular signaling in quorum sensing [46]. Llena et al. [47] examined the effect of the *Streptococcus dentisani* 7746 supernatant on the growth of bacteria implicated in root canal infections. Resistance to the antimicrobial effect of the *S. dentisani* supernatant was detected in the *Sch. odontolytica* AM98a strain. The antimicrobial and antibiofilm activity of *S. salivarius* K12, either of the strain itself or of the supernatant, has not been previously studied in *Sch. odontolytica* and *E. cloacae*.

## 4. Materials and Methods

### 4.1. Bacterial Strains and Culture Conditions

The bacterial strain of *S. mutans* Clarke (ATCC^®^ 35668™) was acquired from the Faculty of Natural Sciences of Comenius University in Bratislava. Potential pathogenic bacteria, including *S. hominis* 41/6, *E. cloacae* 4/2, and *Sch. odontolytica* P10, were isolated from the dental biofilm and teeth extracted from humans with periodontitis. The test strains were grown on blood agar under aerobic conditions, except *Sch. odontolytica* P10, which requires anaerobic conditions (BBL GasPakTM Plus, Becton, Dickinson and Co., Sparks, MD, USA). The blood agar was prepared as Tryptone Soya Agar (HiMedia, Mumbai, India) supplemented with 5% sterile horse blood. The inoculated agar plates with *S. mutans* Clarke were incubated for 48 h at 37 °C. The culture conditions of *S. hominis* 41/6 and *E. cloacae* 4/2 were 24 h at 37 °C, while those of *Sch. odontolytica* P10 were 72 h at 37 °C.

*S. salivarius* K12 was obtained from the commercially available oral probiotic preparation Bactoral (Pharmaceutical Biotechnology, Czech Republic). One tablet was dissolved in 50 mL of brain heart infusion (BHI) broth (pH 7.4; HiMedia Laboratories, Mumbai, India) in a Falcon conical centrifuge tube and incubated for 24 h at 37 °C. Then, 100 μL of the broth was inoculated in blood agar, followed by further incubation for 24 h at 37 °C aerobically.

*S. hominis* 41/6, *E. cloacae* 4/2, *Sch. odontolytica* P10, and *S. salivarius* were identified by PCR for the variable region of the 16S rRNA gene with universal primers [48]. Based on studies to identify *S. salivarius*, PCR analysis was used for the *gtf* gene encoding glucosyltransferase production [49] and the genes encoding salivaricin A [50] and B [51] with specific primers. DNA from *S. salivarius* K12 was isolated using DNAzol^®^ Direct (Molecular Research Center Inc., Cincinnati, OH, USA) according to the manufacturer’s instructions. The amplification products were sent for purification and sequencing (Microsynth, Wien, Austria).

### 4.2. Preparation of the nCFS of S. salivarius K12

The preparation of the CFS of *S. salivarius* K12 was carried out according to the method described by Lin et al. [52]. Briefly, the strain was inoculated on BHI agar (pH 7.4; HiMedia Laboratories, Mumbai, India) supplemented with 1% glucose. The inoculated agar plates were incubated under aerobic conditions for 24 h at 37 °C. A standardized suspension of *S. salivarius* K12 was prepared by resuspending solitary colonies in 5 mL of sterile 0.9% NaCl solution and then adjusting to 1 McFarland turbidity (McF). BHI broth (pH 7.4; HiMedia Laboratories, Mumbai, India) supplemented with 1% glucose was inoculated with a strain suspension (2% inoculum) and incubated for 18 h at 37 °C. The bacterial cells were centrifuged at 4 °C and 4500 RPM for 60 min. The obtained CFS was neutralized with 10 M NaOH to pH 7 (elimination of the effect of low pH) and filtered through a syringe filter with a 0.22 μm pore size (Minisart^®^ Syringe Filter; Sartorius Stedim Biotech, Göttingen, Germany). Subsequently, the nCFS was lyophilized and stored at −70 °C.

### 4.3. In Vitro Assay for Antibiofilm and Antimicrobial Activity of the nCFS

The lyophilized nCFS of *S. salivarius* K12 was resuspended in BHI broth to a concentration of 120 mg/mL. The nCFS, a volume of 180 μL, was added to the first wells of a polystyrene microtiter plate (Greiner ELISA 8 Well Strips, 350 μL, flat bottom, medium binding; Cruinn Diagnostics Ltd., Dublin, Ireland) containing 180 μL of BHI broth and serially diluted, each time by half, using a multipipette, to prepare concentrations of 60 to 7.5 mg/mL. A standardized inoculum of selected potentially pathogenic strains was then inoculated (1 McF, 20 μL). These strains were selected for the study based on biofilm formation. BHI broth with sterile 0.9% NaCl solution and BHI broth with various concentrations of the nCFS were used as negative controls. BHI broth with potentially pathogenic strains without the nCFS was used as a positive control. Microtiter plates with *E. cloacae* 4/2 and *S. hominis* 41/6 were incubated for 24 h at 37 ° C under aerobic conditions and with *S. mutans* Clarke for 48 h at 37 ° C aerobically. Microtiter plates with *Sch. odontolytica* P10 were incubated for 72 h at 37 ° C under anaerobic conditions (BBL GasPakTM Plus; Becton, Dickinson and Co., Maryland, USA). To evaluate the growth of the strains in the presence of various concentrations of the nCFS, the optical density at 570 nm was measured after incubation (SynergyTM 4 Multi-Mode Microplate Reader; BioTek, USA).

The contents of the wells were aspirated, and the biofilm formed was quantified using the crystal violet assay, as described previously [53]. The wells were washed with deionized water and, after drying, stained with 200 μL of 0.1% crystal violet solution for 30 min at room temperature. This was followed by washing again with deionized water and drying, and the biofilm-bound dye was extracted in 200 µL of 10% glacial acetic acid. The optical density was measured at 550 nm (SynergyTM 4 Multi-Mode Microplate Reader; BioTek, USA). Strains were tested in at least three independent experiments, each with 8 replicates. The results were interpreted as the arithmetic mean of the measured values ± standard deviation. The percentage inhibition of growth or biofilm formation was calculated according to the formula described in a study by Jadhav et al. [54]. A_CFS_ represents the absorbance of the well with the test strain and the nCFS and A_o_ the absorbance of the well with the test strain without the nCFS.

Percentage inhibition = [1 − (A_CFS_/A_o_)] × 100

### 4.4. Statistical Analysis

The results of antimicrobial and antibiofilm activity were evaluated using one-way analysis of variance (ANOVA) with an additional Dunnett’s test in the statistical software GraphPad Prism 6.01 (GraphPad Inc., San Diego, CA, USA).

## 5. Conclusions

The nCFS of *S. salivarius* K12 significantly reduced growth in *Streptococcus mutans* Clarke and in *Staphylococcus hominis* 41/6. Biofilm formation decreased in *Schaalia odontolytica* and in *Enterobacter cloacae* 4/2. In *Schaalia odontolytica* P10 and *Enterobacter cloacae* 4/2, the nCFS had no effect on their growth. Further studies will require elucidation of the mechanism of the antibiofilm effect at the molecular level as well, focusing on the evaluation of changes in the expression of genes responsible for the synthesis of bacterial factors involved in the biofilm formation process.
